# Supplementary material for: Analyzing the impact of spatial centrality and courtyard diversity on tourist attractions in the walled city of Lefkoşa
Source: PLoS One. 2025 Aug 22;20(8):e0330956. doi: 10.1371/journal.pone.0330956 (PMC12373240; doi:10.1371/journal.pone.0330956)
Supplement: S1 Data — (ZIP) [file pone.0330956.s001.zip › Minimal Data Set/Spatial Autocorrelation Report_AvG 30.html]

 Spatial Autocorrelation Report

# Spatial Autocorrelation Report

|  |  |  |
| --- | --- | --- |
| Moran's Index | -0.091095 |  |
| z-score | -0.577109 |  |
| p-value | 0.563866 |  |

Given the z-score of -0.577109, the pattern does not appear to be significantly different than random.

Global Moran's I Summary

|  |  |
| --- | --- |
| Moran's Index | -0.091095 |
| Expected Index | -0.032258 |
| Variance | 0.010394 |
| z-score | -0.577109 |
| p-value | 0.563866 |

Dataset Information

|  |  |
| --- | --- |
| Input Feature Class: | Data |
| Input Field: | AV30 |
| Conceptualization: | INVERSE\_DISTANCE |
| Distance Method: | EUCLIDEAN |
| Row Standardization: | True |
| Distance Threshold: | 132.2185 meters |
| Weights Matrix File: | None |
| Selection Set: | False |
