# Supplementary figures and images for: Analyzing the impact of spatial centrality and courtyard diversity on tourist attractions in the walled city of Lefkoşa
Source: PLoS One. 2025 Aug 22;20(8):e0330956. doi: 10.1371/journal.pone.0330956 (PMC12373240; doi:10.1371/journal.pone.0330956)

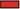

Supplement: S1 Data — (ZIP) [file pone.0330956.s001.zip › Minimal Data Set/Spatial Autocorrelation Report Straighness_files/clusteredBox01.png]

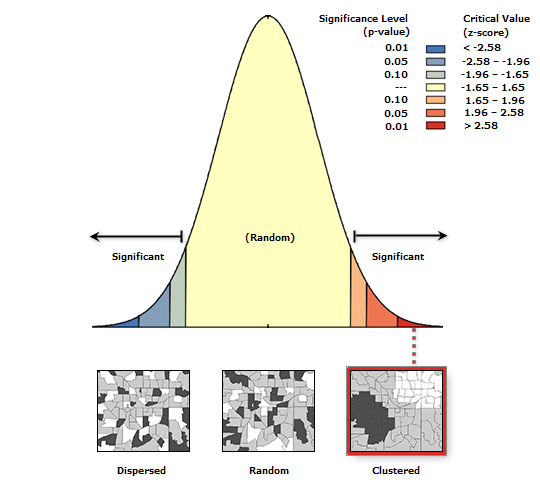

Supplement: S1 Data — (ZIP) [file pone.0330956.s001.zip › Minimal Data Set/Spatial Autocorrelation Report Straighness_files/clusteredValues01.png]

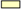

Supplement: S1 Data — (ZIP) [file pone.0330956.s001.zip › Minimal Data Set/Spatial Autocorrelation Report_AvG 29_files/randomBox.png]

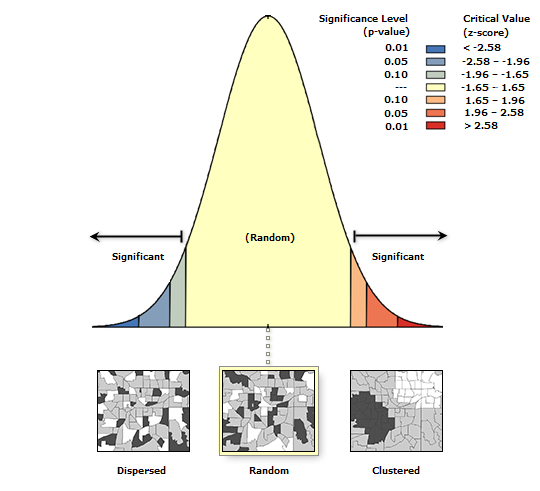

Supplement: S1 Data — (ZIP) [file pone.0330956.s001.zip › Minimal Data Set/Spatial Autocorrelation Report_AvG 29_files/randomValues.png]
