# Supplementary material for: Analyzing the impact of spatial centrality and courtyard diversity on tourist attractions in the walled city of Lefkoşa
Source: PLoS One. 2025 Aug 22;20(8):e0330956. doi: 10.1371/journal.pone.0330956 (PMC12373240; doi:10.1371/journal.pone.0330956)
Supplement: Attachment — Submitted filename: Response_to_Reviewers.docx [file pone.0330956.s003.docx]

**Reviewer one**

**Reviewer Comment 1:**
“The introduction would benefit from explicitly framing the research questions and analytical framework.”

**Response and Suggested Revision: added to P2 line 63**
This study addresses two central research questions:
(1) How does spatial centrality within the street network affect tourist distribution and movement in Lefkoşa’s old city?
(2) How does the diversity of courtyard activities influence tourist engagement across varying levels of spatial centrality?
To answer these questions, the study adopts a mixed-methods analytical framework integrating GIS-based network analysis (PageRank and Straightness centrality) with field observations and regression modeling, offering a spatial and behavioral understanding of urban tourist dynamics.

**Reviewer Comment 2:**
“Some sections like Methodology and Results are overly descriptive or repetitive… grammar issues and awkward phrasing (e.g., 'The research used to type of spatial centrality…')”

**Response and Suggested Revision: P6 l 285**
**Revised:** This research employed two types of spatial centrality measures—PageRank and Straightness—to evaluate the influence of network structure on tourist flow.

Additionally, repetitive statements between sections, such as the methodology and results, were revised to reduce redundancy.

**Reviewer Comment 3:**
“The GLR results indicate a negative relationship between PageRank/straightness and tourist density, which appears counterintuitive…”

**Response and Suggested Revision:** **Addition to Discussion Section p16 l 446:**
“The observed negative relationship between centrality metrics and tourist density may reflect hidden contextual factors in the heritage city. Many highly central streets in Lefkoşa serve as transition corridors with limited visual or functional access to courtyards, which are often concealed behind façades. Conversely, less central streets may host courtyards that are more accessible, visibly promoted, or culturally vibrant, thereby attracting more sustained visitor engagement. This suggests that spatial prominence alone does not guarantee tourist attraction unless paired with accessible and active uses.”

**Reviewer Comment 4:**
“The literature review… spends significant space discussing PageRank in biomedical and digital networks. This could be shortened…”

**Response and Suggested Revision p 16 l 118**:

“While PageRank originated in web analysis and found applications in fields such as epidemiology and bioinformatics [references], its relevance to urban planning lies in its ability to assess indirect influence in complex networks. In urban studies, PageRank has been used to identify critical nodes in transportation and street networks, offering insights into pedestrian flows and accessibility [Agryzkov et al., 2012; Porta et al., 2006].”

This revision refocuses the review on urban planning while briefly acknowledging interdisciplinary roots.

**Reviewer Comment 5:**
“Figures referenced (e.g., Fig. 4–5) are not included or described in sufficient detail…”

**Response and Suggested Revision:** **Revised Caption for Fig 4 p14 l 388:**
**Fig 4. Centrality Analysis Results.**
(A) PageRank Centrality: Streets with higher scores indicate greater influence within the urban network. (B) Straightness Centrality: Areas with higher values indicate more direct access across the network. The color gradient (blue to red) reflects increasing centrality values. These maps are overlaid with courtyard locations for spatial comparison.

**Reviewer 2**

**Reviewer Concern 1:** Why PageRank and Straightness, and how do they relate to pedestrian/tourist movement better than other metrics?

**Response and Suggested Addition p5 l165:**

PageRank was selected for its ability to identify not only directly connected nodes but also those that are indirectly influential through the broader network, capturing hidden patterns of pedestrian importance often missed by simple degree or closeness measures [Agryzkov et al., 2012; Porta et al., 2006]. In dense urban heritage settings like Lefkoşa, this capacity is crucial because street visibility and prominence are shaped by both immediate and global network positions. Straightness centrality, on the other hand, reflects the geometric efficiency of paths and aligns well with pedestrian preferences for direct and intuitive routes, especially in walkable environments [Sevtsuk & Mekonnen, 2012]. Unlike betweenness, which emphasizes through-traffic, or closeness, which favors centralized locations, Straightness offers insights into perceived spatial legibility, a key factor in how tourists navigate historical areas.

**Reviewer Concern 2:** Lack of clarity on how courtyard diversity was defined or quantified.

**Response and Suggested Revision p12 l 362:**

In this study, courtyard “diversity” refers to the range and flexibility of functions supported within a courtyard space. Each courtyard was evaluated using a field survey checklist that captured:
(1) the number of distinct activity types observed (e.g., dining, resting, shopping, cultural events),
(2) the degree of temporal variation in usage (e.g., morning cafés vs. evening performances),
(3) user engagement patterns (e.g., passive vs. interactive), and
(4) physical adaptability (e.g., movable furniture, shaded vs. unshaded zones).
Each courtyard received a diversity score (1–5) based on a composite index summarizing these indicators. This score was used to compare courtyard performance across different spatial centrality zones.

**Reviewer Concern 3:** No discussion of overdispersion or spatial autocorrelation.

**Response and Suggested p 14 l 391**

Before model fitting, we tested for overdispersion by comparing the variance and mean of the count data. The dispersion parameter exceeded 1.5, indicating moderate overdispersion. Therefore, a negative binomial regression was also tested, but the results were consistent with the Poisson GLR. Since the overdispersion was not severe, we retained the Poisson model for interpretability and consistency.

**Spatial Autocorrelation of Residuals p 13 l 379:**

To assess spatial dependence, Moran’s I was calculated on the residuals of the GLR model. The result was near zero (I = -0.015, p > 0.5), indicating no significant spatial autocorrelation and confirming the residuals were randomly distributed.

**Interpretation of Negative PageRank Coefficient p 14 l 404:**

The counterintuitive negative relationship between PageRank and tourist density may be due to the functional nature of central streets. In Lefkoşa, many high-PageRank streets are used for transit or administrative/commercial purposes and lack direct access to culturally active courtyards. Tourists may bypass these streets in favor of more secluded or atmospheric areas with visible and engaging courtyard activities. This suggests that spatial prominence alone does not equate to tourist attractiveness without supporting cultural or experiential value.

**Reviewer Concern 4:** Expand beyond Lefkoşa and connect to global urban tourism or heritage contexts.

**Response and Suggested Addition p 17 l 490**

10.	These findings extend beyond Lefkoşa and are relevant to other walled cities and historic urban districts—such as Fez, Dubrovnik, or Mdina—where the juxtaposition of spatial centrality and hidden cultural nodes like courtyards shapes the visitor experience[54, 55] . In such cities, integrating courtyard activation strategies with spatial accessibility planning can enhance tourist dispersal and engagement while avoiding over-concentration at iconic sites. Similar studies using network analysis in Córdoba (Spain) and Cairo (Egypt) have shown that spatial interventions in secondary streets and semi-private spaces improve the experiential depth of heritage tourism [55, 56]. Urban planners and heritage managers can apply this model to revitalize underused areas, promoting both conservation and sustainable tourist flow

Minor Comments from Reviewer Two

**Reviewer Concern 1:** "Day 29" is unclear; sentences in Methods and Results are long or ambiguous.

**Response and Action Plan p7 l 252:**

**Clarify “Day 29”** early in the *Methods > Tourist Behavior Data* section:

“For consistency, pedestrian activity was recorded on two specific dates: May 29 (referred to as Day 29) and May 30 (Day 30). These represent a weekday and a weekend observation period, respectively.”

**Revise unclear or run-on sentences. Example :**“By using the GLR results Table 2, it is clear that in Day 29 PAGERANK stands out as a significant predictor…”

**Revised p 14 l 396 :**

“As shown in Table 2, PageRank centrality was a significant predictor on May 29 (Day 29). Specifically, each one-unit increase in PageRank was associated with a decrease of approximately 8.7 tourist visits.”

Grammarly software and style checks are used to support formal proofreading.

**Reviewer Concern 2:** Figures are under-explained; captions lack interpretive detail.

**Response and Action Plan:** **Explicitly reference each figure in-text with interpretation. Example:** **In Results (after GLR findings) p 14 l 388:**

“As shown in Figure 4A, high-PageRank segments are concentrated near commercial corridors, while tourist density is more prominent in the areas shown in Figure 2B and 2F. This spatial misalignment explains the negative correlation observed in the regression model.”

**Revise figure captions to explain what the reader should observe.**

**Example revision of Fig. 4 caption p 14:**

**Figure 4. Spatial Centrality Metrics.**
(A) PageRank centrality values across Lefkoşa’s old city: darker red lines indicate higher network influence.
(B) Straightness centrality values: higher values highlight areas with more direct path connections.
These maps help identify how spatial centrality aligns—or diverges—from areas of tourist activity.

**Reviewer Concern 4:** Inconsistent use of “PageRank,” unformatted equations, and citation style issues.

- **Correct capitalization of PageRank and Straightness throughout, DONE** : Perform a “Find and Replace” to ensure uniform formatting:
  - “pagerank” → “PageRank”
  - “straightness” → “Straightness centrality” (when used as a proper metric)
- **Format equations in italic or math mode. Example DONE:**

Change:

- “μi = exp(β0 + β1X1i + … + βkXki)”

To:

*μi* = exp(β₀ + β₁X₁ᵢ + … + βₖXₖᵢ)

- **Ensure all variables are defined at first use DONE.** For example, when introducing *PR(i)*, define it as:

“PR(i) denotes the PageRank score of node *i*, reflecting its relative importance in the network.”

- **Citation check for PLOS ONE DONE:**
  - Confirm in-text references match those in the reference list (1:1 correspondence).
  - Use Vancouver style (numbered citations in square brackets, e.g., [1], [2–4]).
  - Ensure punctuation and spacing around citations follow PLOS formatting, e.g., “in previous studies [3, 5].”
